# Analysis of the prevalence of subtypes of angioedema without urticaria in a reference center in Rio de Janeiro – Brazil

**DOI:** 10.1186/1939-4551-8-S1-A39

**Published:** 2015-04-08

**Authors:** Renata Silva Fernandes, Sérgio Duarte Dortas, Cristiane Fernandes Moreira, Maria Luiza Oliva Alonso, Bruno Emanoel Carvalho Oliveira, Soloni Afra Pires Levy, Alfeu Tavares França, Alfeu Tavares França, Solange Oliveira Rodrigues Valle

**Affiliations:** 1 Hospital Universitário Clementino Fraga Filho Hucff-Ufrj, Brazil; 2 Universidade Iguaçu, Brazil; 3 Hospital São Zacharias, Brazil

## Background

Angioedema is a highly heterogeneous group of conditions and is characterized by sudden, pronounced swelling of the lower dermis and subcutaneous. Because of its frequent coexistence with urticaria, it is often classified in the same manner as urticaria. However, it also includes categories not associated with urticaria. Angioedema without urticaria is characterized by hereditary and acquired angioedema and histaminergic and nonhistaminergic angioedema. The prevalence of subtypes of angioedema without urticaria was estimated at the Clinical Immunology outpatient service of an University Hospital, in Rio de Janeiro.

## Methods

We have classified 118 (40 males and 78 females) outpatients with angioedema without urticaria in categories: hereditary angioedema (HAE), angiotensin-converting enzyme (ACE) inhibitor-induced angioedema and idiopathic angioedema.

## Results

HAE was showed in 98 of 118 patients (83%). ACE inhibitor induced-angioedema was showed in 16 patients (13,5%) and 4 patients (3,5%) were diagnosed with idiopathic angioedema, after a complete investigation of all causes of angioedema.

## Conclusions

In our casuistic, HAE was the most prevalent type of angioedema without urticaria, as described in the literature. However, the prevalence of ACE inhibitor-induced angioedema found in our study was higher, since the reported incidence of this condition ranges from 0,1 to 6%. Correct diagnosis of the subtypes of angioedema without urticaria should be the basis of better understanding and the treatment of these conditions.

